# Development and Validation of a Capillary Zone Electrophoresis–Tandem Mass Spectrometry Method for Simultaneous Quantification of Eight β-Lactam Antibiotics and Two β-Lactamase Inhibitors in Plasma Samples

**DOI:** 10.3390/ph17040526

**Published:** 2024-04-19

**Authors:** Ivana Cizmarova, Peter Mikus, Martin Svidrnoch, Juraj Piestansky

**Affiliations:** 1 Department of Pharmaceutical Analysis and Nuclear Pharmacy, Faculty of Pharmacy, Comenius University in Bratislava, Odbojarov 10, SK-832 32 Bratislava, Slovakia; ivana.cizmarova@fpharm.uniba.sk (I.C.); mikus@fpharm.uniba.sk (P.M.); 2 Toxicological and Antidoping Center, Faculty of Pharmacy, Comenius University in Bratislava, Odbojarov 10, SK-832 32 Bratislava, Slovakia; 3 AGEL Lab, Revolucni 2214/35, CZ-741 01 Novy Jicin, Czech Republic; martin.svidrnoch@lab.agel.cz; 4 Department of Galenic Pharmacy, Faculty of Pharmacy, Comenius University in Bratislava, Odbojarov 10, SK-832 32 Bratislava, Slovakia

**Keywords:** capillary zone electrophoresis, tandem mass spectrometry, β-lactam antibiotics, bioanalysis, therapeutic drug monitoring

## Abstract

Monitoring plasma concentrations of β-lactam antibiotics is crucial, particularly in critically ill patients, where variations in concentrations can lead to treatment failure or adverse events. Standardized antimicrobial regimens may not be effective for all patients, especially in special groups with altered physiological parameters. Pharmacokinetic/pharmacodynamic (PK/PD) studies highlight the time-dependent antibacterial activity of these antibiotics, emphasizing the need for personalized dosing. Therapeutic drug monitoring (TDM) is essential, requiring rapid and accurate analytical methods for precise determination of drugs in biological material (typically plasma or serum). This study presents a novel capillary zone electrophoresis–tandem mass spectrometry (CZE-MS/MS) method designed for the simultaneous quantification of five penicillin antibiotics, two cephalosporins, one carbapenem, and two β-lactamase inhibitors in a single run. The method involves a simple sample pretreatment—precipitation with organic solvent—and has a run time of 20 min. Optimization of CZE separation conditions revealed that 20 mM ammonium hydrogen carbonate (NH_4_HCO_3_) serves as the optimal background electrolyte (BGE). Positive electrospray ionization (ESI) mode, with isopropyl alcohol (IP)/10 mM ammonium formate water solution (50/50, *v*/*v*) as the sheath liquid, was identified as the optimal condition for MS detection. Method validation according to the Food and Drug Administration (FDA) guideline for development of bioanalytical methods demonstrated satisfactory selectivity, linearity, recovery, robustness, and stability. The method’s practicality was evaluated using the Blue Applicability Grade Index (BAGI), yielding a score of 77.5. Moreover, the greenness of the proposed method was evaluated by two commonly used metric tools—Analytical GREEnness (AGREE) and Green Analytical Procedure Index (GAPI). The developed CZE-MS/MS method offers a practical and reliable approach for quantifying a broad spectrum of β-lactam antibiotics in plasma. Its ability to simultaneously quantify multiple analytes in a single run, coupled with a straightforward sample pretreatment, positions it as a valuable and prospective tool for TDM in critically ill patients.

## 1. Introduction

Monitoring plasma concentrations of β-lactam antibiotics (ATBs) is crucial, especially in critically ill patients, where variations in these concentrations can be significant, ranging from subtherapeutic to potentially toxic levels [1]. Low drug concentrations may result in treatment failure, leading to prolonged hospitalization or even death. Conversely, high plasma concentration levels pose the risk of rare but serious adverse events, such as neurotoxicity and nephrotoxicity, which can be dose-dependent and challenging to detect promptly in the intensive care unit (ICU) [2].

Clinical trials often tailor antimicrobial regimens for the “average patient”. However, such a standardized approach may not represent an effective therapeutic management for all individuals [3]. A good example, is critically ill patients in particular, who are very often affected by receiving insufficient ATB treatment under such regimens [4]. Special patient groups, including critically ill, obese, or older individuals, typically exhibit an altered volume of distribution, protein binding, clearance, and other pathophysiological changes, making the prediction of antibiotic concentrations challenging [5]. Additionally, new information indicates a possible correlation between the clinical outcomes of critically ill patients and the serum concentrations of β-lactam ATBs [6].

Pharmacokinetic/pharmacodynamic (PK/PD) studies of these ATBs consistently indicate that their antibacterial activity is time-dependent [7,8,9,10]. It means that the time interval during which plasma concentration of ATBs remains above the minimal inhibitory concentration (MIC) between two administered doses correlates well with treatment effectiveness. Some studies demonstrated that the optimal bactericidal activity is also attained when the plasma concentrations of ATBs exceed four to five times the MIC for 70% to 100% of the dosing interval [11,12,13,14]. Only 60% of ICU patients achieve these PK/PD targets, and not achieving them is associated not only with therapeutic failure and prolonged hospital stay, but also with increased microbial resistance [15]. Therefore, the implementation of therapeutic drug monitoring (TDM) for β-lactam ATBs is imperative. This involves the precise and prompt measurement of the plasma concentration of drugs, enabling tailored dosage adjustments for individual patients. The key requirement is an analytical method that is rapid, reliable, accurate, and robust. There are no immunoassays available for β-lactam ATBs quantification. Liquid chromatography (LC) methods coupled with ultraviolet (UV) [16,17,18,19,20] or mass spectrometry (MS) detection [1,21,22,23,24] are primarily utilized for this purpose. However, a TLC method was also developed for this purpose [25].

Capillary electrophoresis (CE) serves as a sustainable alternative to chromatographic methods, attributed to its minimal sample consumption and avoidance of organic solvents. Capillary zone electrophoresis (CZE) or micellar electrokinetic chromatography (MEKC) methods have been established for β-lactam ATBs quantification, which have predominantly employed UV detection [26,27,28,29,30,31,32,33,34,35,36,37,38,39,40,41] and, in a few cases, also conductivity detection [42,43,44]. Most CE methods are typically designed to quantify only one or two analytes. Even those capable of quantifying multiple analytes (typically 4–6) usually focus on analytes belonging to the same chemical group. For instance, Gáspár et al. developed a MEKC method for the analysis of four cephalosporins [31], Andrási et al. introduced a method for quantifying six cephalosporins [35], and Pham et al. focused on determination of four carbapenems [42]. Similarly, Slampova et al. presented a MEKC method for quantifying four penicillin ATBs recently [38]. A complex overview of CE-based methods used in the analysis of β-lactam ATBs and β-lactamase inhibitors in plasma or serum samples (matrices used for TDM) is present in Table 1. Although the previous CE methods have brought advances in the field of electrodriven separation techniques, the clinical practice is a specific field that demands reliable methods capable of quantifying a broad spectrum of β-lactam ATBs.

**Figure 1:**
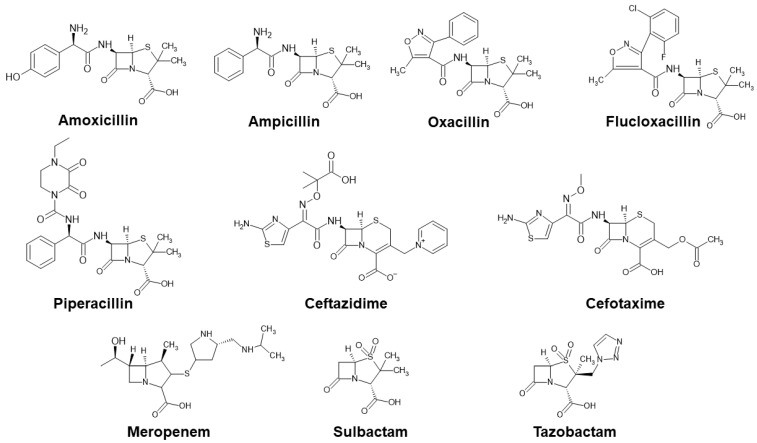
Chemical structures of investigated ATBs.

In this study, we aim to present the pioneering CZE-MS/MS method designed for simultaneous quantification of five penicillins (amoxicillin—AMX, ampicillin—AMP, flucloxacillin—FLX, oxacillin—OXA, piperacillin—PIP), two cephalosporins (cefotafime—CTX, ceftazidime—CAZ), one carbapenem (meropenem—MER), and two β-lactamase inhibitors (sulbactam—SUL, tazobactam—TAZ) in plasma matrix within a single run. Different chemical structures of the investigated analytes (Figure 1) did not pose problems during development of the method. However, some compromises were taken into consideration. The method incorporates a straightforward sample pretreatment, a simple one-step protein precipitation with the use of a convenient organic solvent. Such an analytical approach could be positively perceived by the scientific and clinical community because its implementation into the TDM of β-lactam ATBs and β-lactamase inhibitors may contribute to understand their concentration–effect relationship, which is currently insufficiently documented in the literature.

## 2. Results and Discussion

Development of the new analytical method based on CZE hyphenated with MS/MS detection for simultaneous determination of ten selected β-lactam ATBs and β-lactamase inhibitors demanded compromises during the optimization procedure. It is a consequence of different chemical structures of the analytes resulting in their various physic-chemical properties. From the CZE point of view, generally low repeatability of this method is the main disadvantage, which significantly prevents its wider application in real clinical analysis, including TDM. Therefore, the main emphasis during the optimization procedure was not only on maximizing the intensity of the analytical signal, but also on achieving reproducible results with minimized relative standard deviation (RSD) values.

The aforementioned decision criteria were applied during the whole optimization procedure comprising: (i) optimization of the CZE separation step; (ii) optimization of the MS detection step (including electrospray ionization, ESI). Three replicates were used per each condition during method optimization.

### 2.1. Optimization of the CZE Separation Conditions

All 10 analytes, i.e., 8 β-lactam ATBs and 2 inhibitors of β-lactamase, are characterized by the presence of a carboxylic acid and β-lactam ring in their structures (for details of individual structures see Figure 1). The presence of acidic functional groups predisposes these analytes to electrophoretic analysis in a basic environment, which results in the formation of ions that are essential for the electrophoretic process [45]. In the CE-UV (or CE-DAD) methods for the analysis of β-lactam ATBs published so far, the use of phosphate or borate buffer as a background electrolyte (BGE) predominates [26,27,28,29,31,33,35,36,38,39]. However, these buffers are not compatible with the MS detection step. Therefore, we focused on testing aqueous solutions of ammonium carbonate, (NH_4_)_2_CO_3_, and ammonium hydrogen carbonate, NH_4_HCO_3_, at different concentrations as BGEs in the development of the CZE-MS/MS method. These BGEs fulfill the required criteria on the CE-MS connection—i.e., volatility and low ion strength.

**Figure 2:**
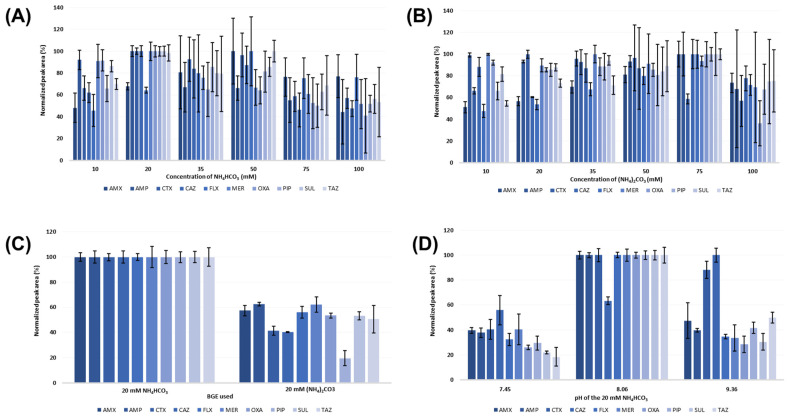
Optimization of BGE for CZE separation of selected β-lactam ATBs and β-lactamase inhibitors. (**A**) Effect of changing NH_4_HCO_3_ concentration on the signal intensity and repeatability. (**B**) Effect of changing (NH_4_)_2_CO_3_ concentration on the signal intensity and repeatability. (**C**) Comparison of obtained signal intensity and repeatability of the investigated analytes using the BGE composed of 20 mM NH_4_HCO_3_ and 20 mM (NH_4_)_2_CO_3_. (**D**) Effect of pH change of the selected BGE (20 mM NH_4_HCO_3_) on the analytical signal intensity and repeatability of the selected analytes. The optimization procedure was performed with the use of ATB standard solutions at the 10 μg·mL^−1^ concentration level.

The selected BGEs were tested in the concentration range of 10–100 mM (see Figure 2A,B). When NH_4_HCO_3_ was tested, optimal results were observed at a concentration of 20 mM. Higher or lower concentrations of the BGE resulted in decreased signal intensity, except of AMX and FLX. Signal intensity maximum of these two analytes was achieved at 50 mM concentration of the BGE. However, significantly higher RSD values of peak area were observed (Figure 2A). On the contrary, in the case of (NH_4_)_2_CO_3_ as BGE, the highest analytical signal of most ATBs was obtained at the concentration of 75 mM. However, excessively high RSD values of the peak area were observed (Figure 2B). This necessitated a compromise between intensity and RSD, and 20 mM (NH_4_)_2_CO_3_ emerged as the optimal concentration. Further, a comparative study between 20 mM NH_4_HCO_3_ and 20 mM (NH_4_)_2_CO_3_ was conducted (see Figure 2C), which confirmed the use of 20 mM NH_4_HCO_3_ as optimal BGE in further experiments. As pH plays a crucial role in the ionization of analytes, a further step in the optimization procedure was focused on investigation of the effect of this parameter on separation properties, resolution, and signal intensity of the ATBs. The original BGE, 20 mM NH_4_HCO_3_, maintained a pH value of 8.06. In our experiments, the pH of the separation environment was adjusted to 7.45 with formic acid, and to 9.36 with 12.5% ammonium hydroxide (NH_4_OH). These pH changes significantly reduced signal intensity for all analytes except for CAZ (see Figure 2D). In general, no significant improvement of the separation and detection properties of the selected ATBs was observed during the experiments with changed pH of the BGE. Therefore, the pH value of 8.06 was selected as the optimal one.

### 2.2. Optimization of MS Detection Conditions

#### 2.2.1. ESI Optimization

The positive ESI mode was selected according to the previously published papers dealing with MS/MS analysis of β-lactam ATBs and inhibitors of β-lactamase [1,21,24,46,47]. Selection of this ESI mode was also accompanied by the basic fact that the sensitivity of the positive ESI mode is significantly higher, as in the case of the negative ESI mode [48,49].

**Figure 3:**
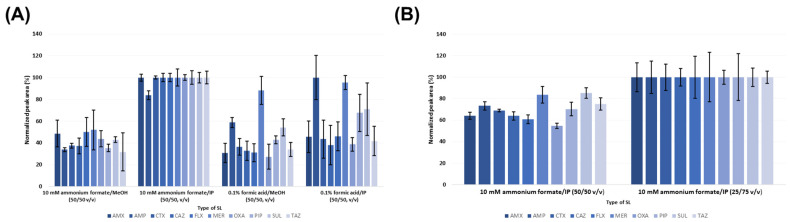
Optimization of the SL composition. (**A**) Effect of SL composition on the analytical signal intensity and reproducibility of investigated β-lactam ATBs and β-lactamase inhibitors. (**B**) Effect of changed organic phase content in the SL mixture on the analytical signal of the investigated molecules. The optimization procedure was performed with the use of ATB standard solutions at the 10 μg·mL^−1^ concentration level.

In the case of the CZE-MS/MS analytical device, the sheath liquid (SL) is a crucial factor of the coaxial sheath flow ESI interface because it ensures adequate ionization of the analyte and provides electrical contact between the liquid in the separation capillary and the electrode [50]. Organic solvents and a water phase enriched with volatile organic acids or their ammonia salts are typically combined to create a sufficient SL. Here, four different types of SL combinations were investigated: (a) methanol (MeOH)/0.1% formic acid water solution (50/50, *v*/*v*), (b) isopropyl alcohol (IP)/0.1% formic acid water solution (50/50, *v*/*v*), (c) MeOH/10 mM ammonium formate water solution (50/50, *v*/*v*), and (d) IP/10 mM ammonium formate water solution (50/50, *v*/*v*), (75/25, *v*/*v*). The main criteria chosen in the optimization procedure were maximization of the analytical signal and its reproducibility. At first, the aforementioned mixtures at 50/50 (*v*/*v*) ratio were tested and compared. According to the results summarized in Figure 3A, the optimal analytical signal properties of all investigated molecules were obtained by using the 10 mM ammonium formate/IP mixture. However, the highest intensity of the analytical signal for AMP was obtained by using the mixture of 0.1% formic acid/IP, but this environment resulted in poor peak area reproducibility (RSD values > 24%).

Typically, increasing the proportion of the organic phase in SL is responsible for improvement of the analytical signal [50]. Therefore, the selected SL composed of 10 mM ammonium formate/IP mixture underwent further investigation involving variation in the inorganic to organic phase ratio, i.e., 25/75 (*v*/*v*). As anticipated, the analytical signal response increased for all tested analytes (Figure 3B), and the increase was within the range of 22–79%. On the contrary, significant worsening of the reproducibility expressed as RSD of peak areas (ranging from 5.8% to 23%) was observed. Due to these facts, the mixture composed of IP/10 mM ammonium formate water solution (50/50, *v*/*v*) was selected and used in further analyses.

Another important ESI parameter is the SL flow rate, which influences the stability and sensitivity of MS detection, as well as the efficacy of the ionization process. During the optimization procedure, the SL flow rate in the range of 4–8 μL min^−1^ was examined. Based on the acquired data, we assessed that the highest analytical signal response was provided at SL flow rate of 6 μL min^−1^. Simultaneously, we observed that with increasing SL flow rate, the RSD of peak areas decreased (this trend was characteristic within all investigated analytes). The resulting RSD of peak areas for SL flow rate set at 6 μL min^−1^ ranged from 5.6% to 17%, while for SL flow rate at 8 μL min^−1^, the RSD values ranged from 1.8% to 4.7%. These results confirm significant improvement of measurement repeatability while maintaining more than 80% signal intensity (except of AMP) compared to the conditions with the achievement of the most intense analytical signal, i.e., SL flow rate of 6 μL min^−1^ (see Figure S1A in Supplementary Materials). According to these findings, we decided to perform a further experiment with the SL flow rate set to 8 μL·min^−1^.

Additional ESI parameters were investigated and optimized in the following ranges: nebulizing gas pressure (5–15 psi), drying gas temperature (150–350 °C), drying gas flow rate (2–13 L·min^−1^), and capillary voltage (3000–5000 V). These parameters are also crucial for a successful ionization process and stability of the analytical signal. The results obtained from the investigation of the aforementioned parameters are summarized in Figure S1B–E. The following values of the ESI parameters were used in further experiments: nebulizing gas pressure—8 psi, drying gas temperature—300 °C, drying gas flow rate—8 L·min^−1^, and capillary voltage—4500 V.

#### 2.2.2. Triple Quadrupole (QqQ) MS Optimization

The next optimization procedures were focused on the QqQ MS/MS instrument, which included employing various QqQ operating modes, such as Scan, Selected Ion Monitoring (SIM), Product Ion, and Multiple Reaction Monitoring (MRM), in a sequential fashion. A methodical application of these modes enabled us to identify relevant precursor (parent) and product (daughter) ions of the investigated β-lactam ATBs and inhibitors of β-lactamase. Additionally, it allowed the selection of characteristic precursor–product ion transitions, ensuring the unambiguous identification and determination of the analytes, along with the establishment of optimal values for parameters like fragmentor voltage and collision energy.

In Scan mode, precursor ions were initially identified, and under the selected conditions, all ten analytes and four internal standards were preferentially singly charged (confirmation was based on the molecular weights of the analytes). The fragmentor voltage was fine-tuned in SIM mode within the 20–200 V range to maximize the intensity of precursor ions. Optimizing the collision energy (tested in the range of 5–20 eV) in the Product Ion mode resulted in obtaining characteristic MS spectra of the analytes and selection of appropriate product ions. The investigated characteristic ions and optimized values of fragmentor voltage and collision energy are summarized in Table 2. For each compound within the MS spectrum, two distinctive product ions were chosen, the qualifier (used for identity confirmation) and the quantifier (an ion with the highest signal intensity).

The unequivocal identification and quantification of the investigated analytes was ensured by applying the MRM mode of the MS detector. The following *m*/*z* ion quantification transitions were applied in the MRM mode for each investigated analyte: AMX 366.1 → 113.7, AMP 350.0 → 106.1, CTX 456 →166.7, CAZ 547.1 → 395.8, FLX 454.1 → 295, MER 384.0 → 113.7, OXA 402.1 → 160.0, PIP 518.0 → 143.0, SUL 234.0 → 123.8, and TAZ 301.1 → 168. These transitions were in good agreement with previously published data [1,22,24,51]. Details regarding quantitation and identity confirmation transitions for the investigated β-lactam ATBs, inhibitors of β-lactamase, and their associated internal standards are summarized in Table 2.

Another parameter that affects the signal-to-noise (S/N) ratio and sensitivity of the MS/MS analyses is the dwell time. It represents a time spent acquiring a specific MRM transition in each MS cycle. The influence of dwell time within the range of 75–200 ms on the signal intensity and S/N ratio of β-lactams was investigated systematically (see Figure S1F in the Supplementary Materials). Increased dwell time resulted in significant S/N ratio decline of all analytes. In contrast, when assessing the analytical signal (peak area), consistent values were observed at dwell times of 75, 100, and 150 ms. The 200 ms setting led to reduced peak areas. According to meticulous evaluation of the obtained data, dwell time of 100 ms represented optimal conditions.

### 2.3. Optimization of Plasma Sample Preparation

Effective pretreatment of biological samples is a crucial element of the whole analysis. Typically, simple and easy procedures are preferred. These criteria fulfill methods based on plasma protein precipitation with organic solvents, which were also used in this work. It is suggested that such simple sample procedures are sufficient enough because the binding of analyzed β-lactam ATBs and inhibitors of β-lactamase to plasma proteins does not exceed 80% [52,53]. Therefore, it is an adequate sample treatment tool before analyzing the total concentration of these selected substances in the blood [54]. However, some problems could arise in case of FLX and OXA because their binding to plasma proteins achieves 95 and 89–94%, respectively. A more detailed focus on sample pretreatment of these two substances in plasma samples is demanded, but the effectiveness of the pretreatment steps in such cases can be assessed and evaluated only according to the data obtained from the large number of real plasma samples from patients hospitalized in the ICU. During the plasma sample preparation optimization procedure, this issue dealing with FLX and OXA was neglected, because the goal of this work was to develop a practical CZE-MS/MS method that would allow simultaneous analysis of a wide range of β-lactams.

The majority of LC-MS/MS methods for ATB determination describe the use of ACN or MeOH as suitable precipitation agents [46,47,55,56]. Our work was focused on testing various precipitation agents, i.e., pure ACN, pure MeOH, and their combinations at various ratios (ACN:MeOH = 3:1, 2:1, 3:2, 1:1) and different volumes (120 µL and 180 µL). Intensity of the analytical signal (expressed as peak area) and its RSD were the main decision criteria during the optimization procedure (see Figure S2A–C in Supplementary Materials). As can be seen, higher content of MeOH in the precipitation mixtures led to decreased intensity of the analytical signal. This tendency was observed for both precipitation mixture volumes, i.e., 120 µL (Figure S2A in Supplementary Materials), and also 180 µL (Figure S2B in Supplementary Materials). Moreover, the use of solely MeOH as precipitating agent was accompanied with an unstable electrical current during the CZE separation, resulting in interruption of the whole analysis. However, the mixture ACN:MeOH at the ratio 3:2 provided improved analytical signal intensity, and the investigated RSD values were relatively high 3.3–15.9%. Higher stability and repeatability of the analytical signal was observed when using mixtures with higher amounts of ACN. The further investigation clearly showed that use of pure ACN at the volume of 120 µL was the optimal precipitating agent (see Figure S2C in Supplementary Materials).

### 2.4. Method Validation

Validation of the developed CZE-MS/MS method for simultaneous determination of eight β-lactam ATBs and two inhibitors of β-lactamase was realized according to the recommendations of the Food and Drug Administration’s (FDA) guideline for bioanalytical method validation [57]. Validation parameters, including selectivity, linearity range, recovery, robustness, the limit of detection (LOD), the lower limit of quantitation (LLOQ), accuracy, precision, carry-over effect, and stability were thoroughly investigated. Table 3, Table 4 and Table 5 summarize the results obtained during the validation procedures.

Selectivity assessment was based on measurements of blank plasma samples, zero-calibrator plasma samples containing only IS, and plasma samples at the first calibration level. The results unequivocally demonstrated the absence of matrix interferents in the migration positions of β-lactam ATBs, inhibitors of β-lactamase, and IS (Figure S3 in Supplementary Materials).

Linearity of the proposed method was evaluated using calibration standards of the selected analytes in the concentration range of 0.5–40 μg·mL^−1^ for AMP, CTX, FLX, and OXA, and in the concentration range of 1–40 μg·mL^−1^ for AMX, CAZ, MER, PIP, SUL, and TAZ (for detailed information, see Section 3.4. Preparation of Standard Solutions). The calibration curves were constructed based on the ratio of analyzed β-lactam ATB or inhibitor of β-lactamase to its corresponding IS (defined in Table 2). Linear calibration curves (correlation coefficient > 0.98) were obtained for the analyzed β-lactam ATBs and inhibitors of β-lactamase. The linearity was approved by the linear regression analysis. Further, calibration equation and standard deviation of the slope and intercept were calculated (Table 3).

The LOD values were determined experimentally, the method based on S/N ratio was applied. LOD values (S/N = 3) for plasma and model water matrices were in the range of 0.009–0.5 μg·mL^−1^ and 0.0009–0.438 μg·mL^−1^, respectively. The LLOQ values were selected as the first points of the calibration curves, representing the lowest concentrations of analytes that can be quantified. Detailed information about individual LOD and LLOQ values for each investigated analyte are summarized in Table 3.

The method’s sensitivity and the achieved LLOQ values play a crucial role in assessing plasma concentrations in critically ill patients in clinical practice. When compared with the therapeutic values reported by Schultz et al. [58], the LLOQ values obtained by our proposed CZE-MS/MS method are adequate for the quantification of the selected ATBs.

**Figure 4:**
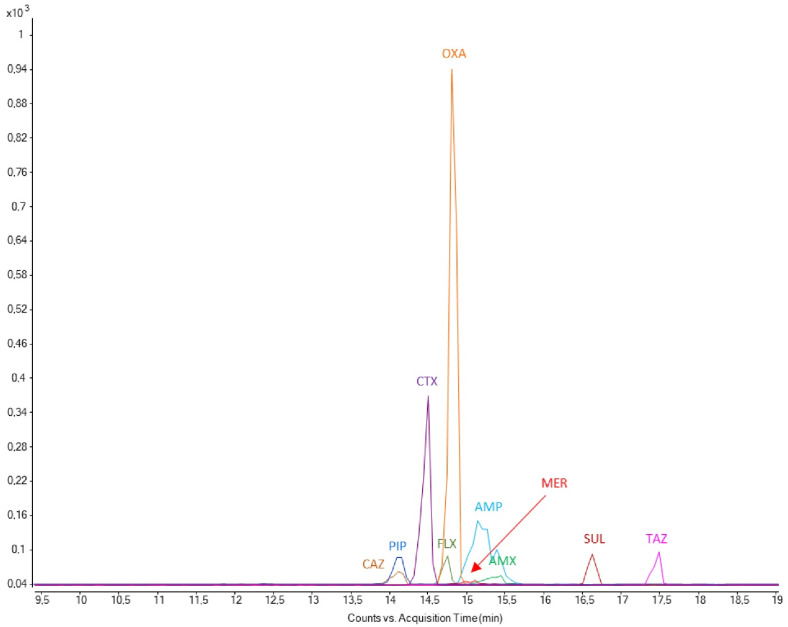
Illustrative extracted Multiple Reaction Monitoring (MRM) electropherogram of investigated β-lactam ATBs and β-lactamase inhibitors at their low QC concentration level.

The precision and accuracy of the developed CZE-MS/MS method were investigated using a series of QC samples prepared at three concentration levels of analytical standards—i.e., 2.5 μg·mL^−1^ (QC low), 15 μg·mL^−1^ (QC medium), and 35 μg·mL^−1^ (QC high), resulting in concrete concentrations of pure antibiotic substances (for detailed information, see Section 3.4. Preparation of standard solutions). Illustrative electropherograms from the analysis of selected β-lactam ATBs and inhibitors of β-lactamase in the QC low sample are present in Figure 4 and Figure S4 in Supplementary Materials.

Precision was examined in terms of repeatability within and between days. The intra-day precision was evaluated by analysis of the QC samples three times in a single day. The inter-day precision was evaluated by repeated analyses of the QC samples over six days (two or three replicates were analyzed per day). The intra-day accuracy (expressed as relative standard deviation as a percentage, % RSD) varied from 1.1 to 12.3%, from 3.3 to 14.6%, and from 4.0 to 14.3% for QC low, QC medium, and QC high, respectively. The corresponding accuracy varied from −14.5 to 18.9%, from −4.5 to 12.6, and from −14.3 to 8.3% for QC low, QC medium, and QC high, respectively. The inter-day accuracy (% RSD) varied from 13.6 to 14.9%, from 6.9 to 13.2%, and from 7.8 to 13.9% for QC low, QC medium, and QC high, respectively. The corresponding accuracy varied from −2.2 to 8.7%, from −3.5 to 6.5, and from −3.9 to 2.6% for QC low, QC medium, and QC high, respectively. All accuracy and precision values for individual analytes and their investigated QC levels are listed in Table 4. The FDA’s requirements for accuracy and precision were satisfied (the determined values ≤ 15%). Only in the case of intra-day accuracy for CAZ at low QC sample did the RSD value exceed the permitted value. Nevertheless, the CZE-MS/MS method can be implemented into reliable quantification of all selected β-lactam ATBs and inhibitors of β-lactamase. This statement is also supported by the fact that the therapeutic plasma concentration of CAZ varies between 20–50 μg·mL^−1^ [49]. Therefore, it is expected that a lower concentration will not play a significant role in the TDM purpose of the developed method.

During the validation process, two types of sample stability were assessed: freeze-to-thaw stability (three cycles of freezing and thawing at room temperature) and short-term stability (sample kept in an autosampler for 24 h at laboratory temperature). The procedure was performed with the use of QC samples at three concentration levels and the determined results were compared with those obtained from the analysis of freshly prepared QC samples. The findings summarized in Table 5 unambiguously confirmed satisfactory short-term and freeze–thaw stability of the analytes under the investigated conditions. The FDA acceptance standard (±15%) was fulfilled by the differences between the nominal and found concentrations, as indicated by the relative error (RE) values (see Table 5).

The validation parameter recovery was evaluated as a ratio of the analytical signals (normalized peak area of the analytes) obtained from the analysis of QC plasma samples spiked with analytical standards at three concentration levels (low, medium, and high) prior to extraction and comparative plasma samples spiked with analytical standards after the extraction process. The recoveries of the investigated β-lactam ATBs and inhibitors of β-lactamase were in the range of 20–40% (see Table S1 in Supplementary Materials). The obtained values are satisfactory because the extraction procedure showed excellent repeatability.

Robustness of the method reflects the effect of small changes in the experimental conditions on analytical results. Here, the effect of a ±1 mM change in BGE concentration was investigated using QC plasma samples. The comparison of results obtained under changed conditions with those ones obtained under standard conditions led to RE values lower than 10%, so the proposed method displayed appropriate robustness.

The carryover effect was assessed by analyzing the highest point of the plasma calibration curve, followed by the analysis of the blank water sample. No presence of peaks was observed in the migration positions of the investigated substances in the blank water sample (Figure S5 in Supplementary Materials).

### 2.5. Method Practicality and Greenness Evaluation

**Figure 5:**
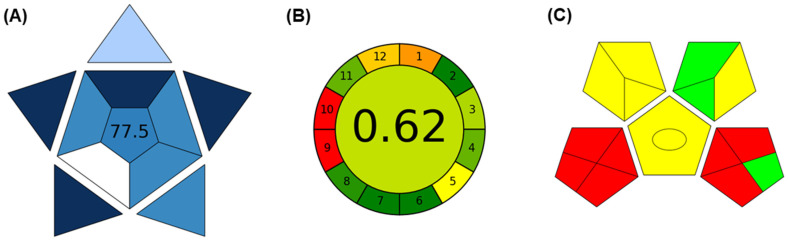
Practicality and greenness evaluation of the proposed CZE-MS/MS method for determination of β-lactam ATBs and β-lactamase inhibitor. (**A**) BAGI evaluation, (**B**) AGREE evaluation, (**C**) GAPI evaluation.

To evaluate the practicality of our developed method, we employed the Blue Applicability Grade Index (BAGI) metric, a new tool used for the assessment of analytical methods [59]. This metric considers ten crucial attributes, including the type of analysis, the simultaneous determination of analytes, sample throughput, reagents and materials consumption, required instrumentation, effectiveness of sample treatment, need for preconcentration, degree of automation, type of sample preparation, and sample amount. According to the input data, a corresponding asteroid pictogram is generated, representing the overall score assigned to the analytical method, ranging from 25 to 100. A score of 25 indicates the poorest performance, while a score of 100 signifies an outstanding performance. A method is deemed practical if it attains a minimum of 60 points. The evaluation of our newly developed method resulted in a score of 77.5 (corresponding pictogram is shown in Figure 5A), which confirmed suitability of the proposed method for its practical use in the area of therapeutic drug monitoring of selected β-lactam ATBs and inhibitors of β-lactamase. The only one limiting step, which significantly decreased the overall practicability of the method (white subsection in the pictogram), is accompanied by the use of sophisticated instrumentation (here, CZE-MS/MS) that is not commonly available in most laboratories.

Moreover, in comparison to some LC analytical approaches, the developed CZE-MS/MS method is characterized by minimization of sample and chemical consumption, with comparable or even better LOD values as some LC-MS/MS methods [23,24]. Similarly, the proposed CZE-MS/MS method does not require any specific demands on sample pretreatment, as is the case of some LC approaches, which demanded, for example, an SPE procedure [23]. Compared to LC-UV approaches [16,17,18,19,20], our approach is characterized by improved selectivity (implementation of MS as a detection technique), which significantly minimizes the risk of interferences with co-medications and endogenous substances. Some of the LC approaches are also characterized by rather long run times and low detection capabilities (UV as the detection technique). Moreover, the demands on chemical consumption are significantly higher than in case of our CE approach.

To clearly present the importance of the developed method as a simple and green analytical method, the greenness of the CZE-MS/MS approach was evaluated by the Analytical GREEnness Metric Approach, AGREE [60], and the Green Analytical Procedure Index, GAPI [61]. Both of the greenness metric tools refer to the 12 SIGNIFICANCE principles of the green analytical chemistry. In the case of the AGREE tool, each of the 12 input variables are transformed into a scale in the 0–1 range. The overall score is shown in the middle of the pictogram, with values close to 1 and dark green color indicating that the assessed procedure is greener [60]. In our case, the overall greenness score obtained with the use of the AGREE metric was 0.62 (Figure 5B). The limiting steps, which decreased the overall score, were associated with the use of instrumentation demanding high energy consumption (red color of the subsection 9 in the pictogram) and no use of reagents obtained from renewable sources (red color of the subsection 10 in the pictogram). In the case of the GAPI greenness evaluation procedure, seven subsections of the pictogram (Figure 5C) were red, especially those ones accompanied with the sample collection, preservation, transport, and storage. Similarly, the sophisticated instrumentation represented by CZE-MS/MS, which demands high energy consumption, was also responsible for presence of more red subsections in the final GAPI pictogram. However, according to the obtained results, the proposed CZE-MS/MS approach represents a practicable and also green analytical approach implementable into the TDM.

### 2.6. Limitations and Future Perspectives of the Study

The developed and validated CZE-MS/MS method for determination of selected β-lactam ATBs represents only a preliminary study and, therefore, it has some limitations. The first significant limitation is the absence of real clinical samples from critically ill patients undergoing ATB therapy. Here, only plasma samples from six healthy volunteers spiked with the analytical standards of investigated ATBs were used. Therefore, to really confirm the application potential of the proposed method, analysis of real clinical samples will be essential. Analysis of a large number of samples is highly demanded. Further challenges are accompanied with more detail and comprehensive investigation of stability of the samples during the whole preanalytical and analytical stage. We expect that this step is critical to obtain reliable data. Testing of appropriate collection tubes and storage conditions of unpretreated and pretreated samples is of great importance, because these steps represent significant sources of analytical errors.

## 3. Materials and Methods

### 3.1. Chemicals and Samples

Merck (Darmstadt, Germany), Sigma Aldrich (Steinheim, Germany), and Fluka (Buchs, Switzerland) provided the LC-MS-grade chemicals required to prepare the electrolyte solutions (i.e., ammonium carbonate—(NH_4_)_2_CO_3_, ammonium hydrogen carbonate—NH_4_HCO_3_), as well as sheath liquid solutions (methanol—MeOH, isopropyl alcohol—IP, formic acid, and ammonium formate). LC-MS-grade acetonitrile (ACN) was obtained from VWR International (Vienna, Austria). Sodium hydroxide (NaOH), p.a. quality, was obtained from Agilent Technologies (Santa Clara, CA, USA). Dimethyl sulfoxide (DMSO), p.a. quality, was obtained from Sigma Aldrich. The electrolytes, sheath liquid, and samples were prepared using demineralized water, which was produced using a Direct-Q^®^ 3 UV water purification system (Millipore, Molsheim, France). Electrolytes were kept in the refrigerator before analysis and filtered using disposable membrane filters with a 0.22 μm pore size from Millipore.

Analytical-grade standards of investigated ATBs (amoxicillin, ampicillin, cefotaxime sodium salt, ceftazidime, flucloxacillin sodium salt, meropenem trihydrate, oxacillin sodium monohydrate, piperacillin sodium salt, sulbactam sodium salt, and tazobactam) were purchased from Sigma Aldrich. The deuterated internal standards ([^13^C, ^2^H_3_]-cefotaxime, [^2^H_6_]-meropenem, [^2^H_5_]-piperacillin sodium salt, and [^13^C_2_, ^15^N_3_]-tazobactam sodium salt) were purchased from Alsachim (Strasbourg, France).

### 3.2. Instrumentation

All CZE-MS/MS experiments were conducted using an Agilent 7100 capillary electrophoresis system (Agilent Technologies) coupled with an Agilent 6410 Series Triple Quadrupole tandem mass spectrometer (Agilent Technologies), featuring a commercial coaxial sheath liquid electrospray (ESI) interface. Separation occurred in a 90 cm × 50 μm inside diameter (ID) bare fused-silica capillary purchased from MicroSolv Technology Corporation (Leland, NC, USA). Sample injection was performed hydrodynamically, lasting 10 s at 50 mbar. Subsequently, a short zone of BGE was hydrodynamically injected for 2 s at 50 mbar pressure to enhance sample quantitative injection and reproducibility.

Experiments were conducted under voltage of +20 kV and normal polarity, resulting in currents of 3–5 μA. The sheath liquid, consisting of IP and a 10 mM ammonium formate water solution (50/50, *v*/*v*), was delivered by an Agilent 1260 Infinity isocratic LC pump (Agilent Technologies) at a flow rate of 8 μL·min^−1^. The MS operated in positive-ion MRM mode, utilizing characteristic precursor ion–product ion mass transitions for each investigated substance. The dwell time, or the period during which MS collects data for a specific MRM transition, was set at 100 ms. Additional MS parameters were configured as follows: capillary voltage—4500 V, nebulizing gas (nitrogen) pressure—8 psi, drying gas (nitrogen) temperature—300 °C, and drying gas (nitrogen) flow—8 L·min^−1^.

### 3.3. Capillary Treatment

A new separation capillary was primed for its initial use by flushing it with a 1 M NaOH aqueous solution for 15 min. Subsequently, the capillary underwent a 10 min preconditioning with BGE and a 15 min rinse with demineralized water, all performed at a pressure of 950 mbar. Prior to each injection, a 2 min flush with BGE and subsequent application of a negative voltage of −25 kV for 30 s was realized to achieve re-equilibration. Post-run capillary treatment included a 2 min rinsing of the capillary with pure MeOH.

To minimize carry-over and enhance analysis repeatability, preconditioning and postconditioning procedures were implemented. At the end of each day, the capillary underwent a 20 min rinse with demineralized water, followed by a 10 min rinse with BGE. The ends of the capillary were soaked in the BGE overnight. All steps were performed at a consistent laboratory temperature.

### 3.4. Preparation of Standard Solutions

To prepare standard solutions, 10 mg of each individual reference standard powder was dissolved in 1 mL of DMSO (amoxicillin, meropenem trihydrate, flucloxacillin sodium salt, and oxacillin sodium monohydrate) or demineralized water (ampicillin, cefotaxime sodium salt, ceftazidime, piperacillin sodium salt, sulbactam sodium salt, and tazobactam) to create individual stock solutions. Each stock solution was aliquoted (50 µL) in 0.2 mL Eppendorf PCR tubes (Eppendorf, Hamburg, Germany) and the aliquots were stored at −80 °C.

The individual reference standard working solutions were prepared from stock solutions by their dilution in demineralized water to obtain the final concentration of 1 mg.mL^−1^. The prepared reference standard working solutions were aliquoted (5 aliquots of 100 µL) in 0.2 mL Eppendorf PCR tubes and stored for a maximum of five days at −20 °C. Simultaneously, individual internal standard (IS) stock solutions were created by dissolving 1 mg of their powder in 1 mL of DMSO, and the 10 µL aliquots were stored at −80 °C. The IS working solution was prepared daily by mixing 7 µL of each IS stock solution with 9972 µL of ACN.

The calibration standards and quality control (QC) standards were prepared by appropriate dilution of the individual reference standard working solutions with demineralized water. These calibrator mixtures were further processed according to the following steps. Each calibration point and QC sample were prepared from the calibrator mixtures by pipetting 30 µL of the demanded calibrator mix into the 1.5 mL Eppendorf tube, followed by adding 30 µL of water (for model aqueous matrices) or plasma (for biological matrices), and 120 µL of the IS working solution, which also served as a precipitating agent. After vortexing and incubating the mixture at room temperature for 10 min, the samples were centrifuged at 30,000× *g* for 10 min, and the supernatant was promptly transferred to a CE vial and directly injected into the CE apparatus. Each sample underwent three measurements. The range of calibration standards, which included AMP, CTX, FLX, and OXA, spanned from 0.5 to 40 µg·mL^−1^ (individual concentrations 0.5, 5, 10, 20, 30, and 40 µg·mL^−1^). For the remaining standards, including AMX, CAZ, MER, PIP, SUL, and TAZ, the calibration range was of 1–40 µg·mL^−1^ (individual concentrations 1, 5, 10, 20, 30, and 40 µg·mL^−1^). Subsequently, the concentrations of the reference standards were recalculated to their pure substances, and the final concentrations of the calibration solutions were in following ranges: 1–40 µg·mL^−1^ for AMX, CAZ, TAZ (individual concentrations 1, 5, 10, 15, 20, 30, 40 µg·mL^−1^); 0.5–40 µg·mL^−1^ for AMP (individual concentrations 0.5, 5, 10, 20, 30, 40 µg·mL^−1^); 0.45–36.38 µg·mL^−1^ for OXA (individual concentrations 0.45, 4.55, 9.09, 18.19, 27.28, 36.28 µg·mL^−1^); 0.48–38.15 µg·mL^−1^ for CTX and FLX (individual concentrations 0.48, 4.77, 9.54, 19.08, 28.62, 38.15 µg·mL^−1^); 0.88–35.06 µg·mL^−1^ for MER (individual concentrations 0.88, 4.38, 8.76, 17.53, 26.19, 35.06 µg·mL^−1^); 0.91–36.55 µg·mL^−1^ for SUL (individual concentrations 0.91, 4.57, 9.14, 18.28, 27.42, 36.55 µg·mL^−1^); and 0.96–38.30 µg·mL^−1^ for PIP (individual concentrations 0.96, 4.79, 9.57, 19.15, 28.72, 38.30 µg·mL^−1^). The QC samples were prepared at three concentration levels, i.e., low, medium, and high. The individual QC samples concentrations for each investigated ATB were as follows: (a) QC low—2.19 µg·mL^−1^ (MER), 2.27 µg·mL^−1^ (OXA), 2.28 µg·mL^−1^ (SUL), 2.38 µg·mL^−1^ (CTX, FLX), 2.39 µg·mL^−1^ (PIP), 2.50 µg·mL^−1^ (AMX, AMP, CAZ, TAZ); (b) QC medium—13.15 µg·mL^−1^ (MER), 13.64 µg·mL^−1^ (OXA), 13.71 µg·mL^−1^ (SUL), 14.31 µg·mL^−1^ (CTX, FLX), 14.36 µg·mL^−1^ (PIP), 15 µg·mL^−1^ (AMX, AMP, CAZ, TAZ); (c) QC high—30.68 µg·mL^−1^ (MER), 31.83 µg·mL^−1^ (OXA), 31.98 µg·mL^−1^ (SUL), 33.38 µg·mL^−1^ (FLX), 33.39 µg·mL^−1^ (CTX), 33.51 µg·mL^−1^ (PIP), 35 µg·mL^−1^ (AMX, AMP, CAZ, TAZ).

Plasma samples used in the experiments were obtained from six healthy volunteers. Pooled plasma samples were used during the whole calibration and validation procedure. The experimental study was conducted according to the guidelines of the Declaration of Helsinki, and approved by the Ethics Committee of the Faculty of Pharmacy Comenius University in Bratislava, Bratislava, Slovakia (protocol code 05/2021, date of approval: 15 December 2021).

## 4. Conclusions

In this research, a pioneering CZE-MS/MS method was developed for the simultaneous quantification of five penicillin ATBs, two cephalosporins, one carbapenem, and two β-lactamase inhibitors in a single run. The method, utilizing a simple sample pretreatment based on plasma protein precipitation using organic solvent, addresses the need for reliable tools capable of quantifying a broad spectrum of β-lactam ATBs. A complex optimization procedure led to excellent results during the validation procedure. A complex analysis of 10 substances was realized within 20 min, which makes the developed method attractive for its implementation in real clinical and bioanalytical laboratories. Moreover, this statement was proved by the BAGI metrics, which confirmed the suitability of the method for TDM of β-lactam ATBs and β-lactamase inhibitors in clinical practice. The findings indicate that this method is a reliable tool for precise and prompt analysis of ATB plasma concentrations, enabling personalized dosage adjustments for critically ill patients. However, there is a need to verify this very promising approach by analyzing large numbers of real clinical samples collected from patients.

## Figures and Tables

**Table 1:** CE methods used in the analysis of β-lactam ATBs and β-lactamase inhibitors in plasma and serum matrices.

Method	Matrix	Sample Preparation	Separation Conditions	LOD (μg·mL^−1^)	Analytes	Reference
MEKC-UV (λ = 210 nm)	plasma	-	uncoated fused silica capillary; ID 50 µm; L_eff_ = 50 cm; BGE = 20 mM phosphate–borate buffer + 50 mM SDS, pH = 8.5	1.3	aspoxicillin	[26]
CZE-UV (λ = 210 nm)	plasma	dilution	uncoated fused silica capillary; ID 50 µm; L_eff_ = 40 cm; BGE = 20 mM phosphate buffer, pH = 6	4	cefodizime	[27]
				2	cefuroxime	
				6	cefpirome	
				2	cefotaxime	
CZE-UV (λ = 254 nm)	plasma	precipitation	uncoated fused silica capillary; ID 75 µm; L_eff_ = 50 cm; BGE = 40 mM borate buffer, pH = 9.2	2	cefotaxime	[28]
MEKC-UV (λ = 254 nm)	plasma	dilution	uncoated fused silica capillary; ID 75 µm; L_eff_ = 50 cm; BGE = 30 mM phosphate buffer + 165 mM SDS, pH = 8	1	deacetylcefotaxime	
CZE-DAD (λ = 200 nm, 303 nm)	plasma	dilution/ precipitation	uncoated fused silica capillary; ID 75 µm; L_eff_ = 72 cm; BGE = 10 mM phosphate buffer, pH = 7.2	4	meropenem	[29]
MEKC-UV (λ = 270 nm)	plasma	ultracentrifugation	uncoated fused silica capillary; ID 50 µm; L_eff_ = 56 cm; BGE = 20 mM citrate buffer + 50 mM SDS, pH = 2.8	0.2	cefpirome	[30]
MEKC-UV (λ = 270 nm)	serum	-	uncoated fused silica capillary; ID 50 µm; L_eff_ = 48.5 cm; BGE = 25 mM borate buffer + 100 mM SDS, pH = 9.2	-	cefuroxime cefotaxime ceftriaxone ceftazidime	[31]
CZE-UV (λ = 270 nm)	plasma	precipitation	uncoated fused silica capillary; ID 50 µm; L_eff_ = 8.5 cm; BGE = 20 mM sodium hydrogen phosphate, pH = 6.4	1	cefazolin	[32]
	microdialysates	dilution		2		
MEKC-UV (λ = 197 nm)	serum	-	uncoated fused silica capillary; ID 75 µm; L_eff_ = 50 cm; BGE = 25 mM borate buffer + 90 mM SDS, pH = 10	2	meropenem	[33]
MEKC-UV (λ = 300 nm)	plasma	SPE	uncoated fused silica capillary; ID 50 µm; L_eff_ = 30 cm; BGE = 40 mM TRIS buffer + SDS, pH = 8	0.2	meropenem	[34]
MEKC-UV (λ = 270 nm)	serum	-	Polyimide-coated silica capillary; ID 50 µm; L_eff_ = 40 cm; BGE = 25 mM borate buffer + 50 mM SDS, pH = 9.1	-	cefazolin cefepime cefamandole cefuroxime ceftazidime ceftriaxone	[35]
CZE-UV (λ = 214 nm)	serum	precipitation	uncoated fused silica capillary; ID 75 µm; L_eff_ = 50 cm BGE = 50 mM tetraborate, pH = 9	5	ceftriaxone	[36]
				1	ceftizoxime	
CZE-UV (λ = 260 nm, 200 nm)	plasma microdialysates	precipitation	INST-coated fused silica capillary; ID 25 µm; L_eff_ = 31.5 cm; BGE = 50 mM chloroacetic acid + 20% MeOH + 0.5% INST, pH = 2.32	0.42	ceftazidime	[37]
CZE-C4D	plasma	SPE	uncoated fused silica capillary; ID 50 µm; L_eff_ = 50 cm; BGE = 10 mM TRIS, pH = 8	0.45	doripenem meropenem imipenem ertapenem	[42]
CZE-C4D	serum microdialysates	precipitation	INST-coated silica capillary; ID 25 µm; L_eff_ = 18 cm; BGE = 0.5 M acetic acid	0.043	amoxicillin	[43]
			INST-coated silica capillary; ID 25 µm; L_eff_ = 18 cm; BGE = 3.2 M acetic acid + 13% MeOH (for ceftazidime)	0.096	ceftazidime	
MEKC-UV (λ = 200 nm)	serum	μ-EME	uncoated fused silica capillary; ID 75 µm; L_eff_ = 39 cm; BGE = 25 mM phosphate buffer + 50 mM SDS, pH = 8.13	0.17	penicillin	[38]
				0.2	phenoxypenicillin	
				0.13	ampicillin	
				0.12	amoxicillin	
MEKC-DAD (λ = 300 nm)	plasma	FESS, sweeping, SPE	uncoated fused silica capillary; ID 50 µm; L_eff_ = 50 cm; high-conductivity buffer = 150 mM phosphate (pH = 2.5) + 20% MeOH; low-conductivity buffer = 50 mM phosphate buffer (pH = 2.5) + 100 mM SDS	0.4	doripenem	[39]
CZE-UV (λ = 215 nm)	plasma	protein precipitation	uncoated fused silica capillary; ID 50 µm; L_eff_ = 40.2 cm; BGE = 15 mM sodium borate buffer (pH 9.3)	0.56 0.95 2.09	Piperacillin Tazobactam cefepime	[40]

Abbreviations: BGE—background electrolyte, C4D—conductivity detection, FESS—field-enhanced sample stacking, ID—internal diameter, L_eff_—effective capillary length, LOD—limit of detection, MeOH—methanol, μ-EME—microelectromembrane extraction, SDS—sodium dodecyl sulphate, SPE—solid-phase extraction, TRIS—2-Amino-2-(hydroxymethyl)propane-1,3-diol.

**Table 2:** Multiple Reaction Monitoring (MRM) conditions for the investigated β-lactam ATBs, inhibitors of β-lactamase, and their internal standards.

Analyte	Parent Ion *m*/*z* [M+H]^+^	Quantifier *m*/*z* [M+H]^+^	Qualifier *m*/*z* [M+H]^+^	Fragmentor Voltage (V)	Collision Energy (eV)	Internal Standard
Sulbactam	234	123.8	141.5	80	15	[^13^C_2_, ^15^N_3_]-tazobactam
Tazobactam	301.1	168	207.1	120	15	[^13^C_2_, ^15^N_3_]-tazobactam
[^13^C_2_, ^15^N_3_]-tazobactam	306	210		120	15	
Ampicillin	350	106.1	191.9	100	15	[^2^H_5_]-piperacillin
Amoxicillin	366.1	113.7	349.1	80	10	[^2^H_5_]-piperacillin
Meropenem	384	113.7	274.8	100	15	[^2^H_6_]-meropenem
[^2^H_6_]-meropenem	390.2	147.2		100	15	
Oxacillin	402.1	160	242.7	100	10	[^2^H_5_]-piperacillin
Flucloxacillin	454.1	295	237.5	140	10	[^2^H_5_]-piperacillin
Cefotaxime	456	166.7	211.1	120	15	[^13^C, ^2^H_3_]-cefotaxime
[^13^C, ^2^H_3_]-cefotaxime	460.1	166.9		120	15	
Piperacillin	518	143	159.9	140	10	[^2^H_5_]-piperacillin
[^2^H_5_]-piperacillin	532.2	148.1		140	10	
Ceftazidime	547.1	395.8	467.6	120	15	[^13^C, ^2^H_3_]-cefotaxime

**Table 3:** Operation and calibration parameters of the CE-MS/MS method for β-lactam ATBs and inhibitors of β-lactamase in model water and plasma samples.

		Calibration Range (µg·mL^−1^)	t_m_ (min), n = 6	RSD_tm_ (%), n = 6	RSD_area_ (%), n = 6	a (Counts)	SD_a_	b (Counts·µg^−1^·mL)	SD_b_	r^2^	LOD (µg·mL^−1^)	LLOQ (µg·mL^−1^)	N
Plasma matrix	AMX	1–40	17.06	0.6	4.8	0.130	0.003	−0.020	0.063	0.992	0.100	1.000	5762
	AMP	0.5–40	16.76	0.3	1.5	0.932	0.031	−0.586	0.514	0.988	0.010	0.500	32,125
	CTX	0.48–38.15	16.02	0.1	9.4	0.312	0.005	0.158	0.114	0.995	0.010	0.477	53,143
	CAZ	1–40	15.57	0.3	9.5	0.050	0.001	0.000	0.023	0.993	0.500	1.000	5860
	FLX	0.48–38.15	16.3	0.2	11.4	0.139	0.004	−0.069	0.063	0.991	0.010	0.477	8772
	MER	0.88–35.06	16.58	0.5	13.5	0.097	0.002	0.078	0.034	0.995	0.438	0.876	2925
	OXA	0.45–36.38	16.44	0.2	2.4	0.730	0.025	−0.023	0.437	0.985	0.009	0.455	20,774
	PIP	0.96–38.30	15.63	0.1	1.5	0.186	0.005	0.089	0.098	0.991	0.010	0.957	7191
	SUL	0.91–36.55	19.74	0.5	7.1	0.581	0.013	−0.218	0.264	0.992	0.091	0.914	51,854
	TAZ	1–40	18.68	0.3	5.8	0.493	0.009	0.199	0.195	0.995	0.050	1.000	40,860
Water matrix	AMX	1–40	21.43	1.1	8.1	0.129	0.005	0.216	0.099	0.987	0.050	1.000	5586
	AMP	0.5–40	21.50	0.6	6.4	0.782	0.020	0.846	0.353	0.993	0.001	0.500	8279
	CTX	0.48–38.15	19.50	0.1	5.1	0.329	0.007	−0.021	0.147	0.993	0.001	0.477	282,724
	CAZ	1–40	18.82	0.2	3.0	0.064	0.002	−0.066	0.030	0.992	0.010	1.000	21,266
	FLX	0.48–38.15	19.96	0.4	3.1	0.125	0.004	0.094	0.067	0.990	0.010	0.477	49,069
	MER	0.88–35.06	20.28	0.1	1.3	0.088	0.003	0.160	0.054	0.991	0.438	0.876	50,658
	OXA	0.45–36.38	20.14	0.4	7.5	0.704	0.021	0.133	0.340	0.992	0.001	0.455	379,984
	PIP	0.96–38.30	18.88	0.1	5.6	0.184	0.005	0.038	0.098	0.991	0.010	0.957	342,736
	SUL	0.91–36.55	25.13	0.1	1.5	0.760	0.021	−0.784	0.383	0.993	0.009	0.914	401,423
	TAZ	1–40	23.53	0.1	4.6	0.470	0.011	0.156	0.202	0.994	0.010	1.000	435,107

**Table 4:** The intra- and inter-day accuracy and precision of the CE-MS/MS method for β-lactam ATBs and inhibitors of β-lactamase in plasma QC samples.

		QC Low				QC Medium				QC High			
		Nominal (µg·mL^−1^)	Found (µg·mL^−1^)	RSD (%)	RE (%)	Nominal (µg·mL^−1^)	Found (µg·mL^−1^)	RSD (%)	RE (%)	Nominal (µg·mL^−1^)	Found (µg·mL^−1^)	RSD (%)	RE (%)
Intra-day, n = 3	AMX	2.50	2.18	6.8	−14.5	15.00	17.17	10.4	12.6	35.00	31.7	12.5	−10.5
	AMP	2.50	2.67	1.6	6.3	15.00	16.04	10.3	6.5	35.00	36.2	14.3	3.4
	CTX	2.38	2.65	8.2	10.2	14.31	15.56	7.0	8.1	33.39	32.8	5.4	−1.8
	CAZ	2.50	3.08	10.7	18.9	15.00	15.28	11.7	1.8	35.00	30.6	7.7	−14.3
	FLX	2.38	2.19	3.4	−8.9	14.31	16.12	5.1	11.3	33.38	31.5	12.0	−5.9
	MER	2.19	2.39	12.3	8.2	13.15	14.19	3.3	7.4	30.68	28.5	5.1	−7.6
	OXA	2.27	2.14	2.4	−6.1	13.64	15.38	13.5	11.3	31.83	30.8	12.1	−3.3
	PIP	2.39	2.84	1.1	15.6	14.36	13.74	7.2	−4.5	33.51	31.4	6.4	−6.8
	SUL	2.28	2.44	8.4	6.2	13.71	13.53	14.6	−1.4	31.98	34.9	11.9	8.3
	TAZ	2.50	2.50	9.9	0.1	15.00	15.78	10.3	4.9	35.00	36.5	4.0	4.1
Inter-day, n = 16	AMX	2.50	2.58	14.0	3.1	15.00	14.73	12.4	−1.8	35.00	35.6	10.5	1.7
	AMP	2.50	2.64	14.3	5.3	15.00	14.55	10.7	−3.1	35.00	34.8	11.8	−0.7
	CTX	2.38	2.39	13.2	1.9	14.31	15.29	10.6	6.5	33.39	32.5	7.8	−2.6
	CAZ	2.50	2.49	14.9	−1.0	15.00	14.58	9.8	−2.9	35.00	34.3	12.0	−1.9
	FLX	2.38	2.51	14.6	6.4	14.31	14.18	13.2	−0.9	33.38	34.2	10.6	2.3
	MER	2.19	2.40	13.6	8.7	13.15	13.78	6.9	4.6	30.68	31.4	9.0	2.3
	OXA	2.27	2.41	14.7	5.6	13.64	14.40	11.7	5.3	31.83	30.6	11.8	−3.9
	PIP	2.39	2.48	14.5	3.4	14.36	13.88	8.0	−3.5	33.51	33.6	8.7	0.4
	SUL	2.28	2.46	13.6	7.1	13.71	13.60	11.7	−0.8	31.98	32.8	13.9	2.6
	TAZ	2.50	2.45	14.0	−2.2	15.00	15.35	7.9	2.3	35.00	35.3	10.0	0.9

**Table 5:** Stability testing of β-lactam ATBs and inhibitors of β-lactamase in plasma QC samples.

		QC Low				QC Medium				QC High			
		Nominal (µg·mL^−1^)	Found (µg·mL^−1^)	RSD (%)	RE (%)	Nominal (µg·mL^−1^)	Found (µg·mL^−1^)	RSD (%)	RE (%)	Nominal (µg·mL^−1^)	Found (µg·mL^−1^)	RSD (%)	RE (%)
Autosampler stability, n = 5	AMX	2.50	2.14	3.3	−1.9	15.00	19.84	5.8	13.5	35.00	36.31	3.3	12.8
	AMP	2.50	2.21	4.6	−4.1	15.00	18.38	11.2	12.7	35.00	41.02	3.4	11.7
	CTX	2.38	2.72	2.5	2.4	14.31	15.93	2.2	2.3	33.39	31.50	3.2	−4.1
	CAZ	2.50	3.29	6.2	6.4	15.00	13.45	3.8	−13.6	35.00	29.09	4.3	−5.3
	FLX	2.38	2.44	11.9	10.4	14.31	16.77	13.5	3.9	33.38	37.00	2.9	14.8
	MER	2.19	2.17	8.7	−10.2	13.15	12.48	9.7	−13.7	30.68	28.30	8.4	−0.7
	OXA	2.27	2.12	12.2	−1.0	13.64	17.83	2.9	13.8	31.83	34.74	4.8	11.2
	PIP	2.39	2.40	3.2	6.7	14.36	13.07	3.9	−5.2	33.51	30.24	5.8	−3.7
	SUL	2.28	2.55	13.0	4.6	13.71	15.25	3.9	11.3	31.98	35.79	11.1	2.5
	TAZ	2.50	2.50	5.6	0.1	15.00	16.76	6.3	5.8	35.00	35.56	12.9	−2.7
Freeze-to-thaw stability, n = 5	AMX	2.50	2.01	9.8	−9.4	15.00	14.12	12.7	−1.4	35.00	34.41	13.6	−6.0
	AMP	2.50	2.14	11.5	0.4	15.00	16.55	7.6	12.9	35.00	35.09	9.3	2.2
	CTX	2.38	1.81	8.9	−14.8	14.31	18.85	6.7	11.6	33.39	27.35	9.2	−12.8
	CAZ	2.50	2.45	13.9	13.6	15.00	17.52	8.7	9.4	35.00	29.80	12.4	−6.3
	FLX	2.38	2.42	12.7	14.7	14.31	14.85	10.2	14.2	33.38	32.38	7.0	−14.0
	MER	2.19	4.25	5.6	−2.3	13.15	16.33	11.4	9.3	30.68	31.56	8.7	3.1
	OXA	2.27	5.32	8.7	−3.7	13.64	16.58	11.4	14.3	31.83	30.80	14.4	8.1
	PIP	2.39	2.09	5.0	2.3	14.36	14.88	3.7	12.9	33.51	27.17	9.3	−13.6
	SUL	2.28	2.31	8.3	14.8	13.71	16.73	4.9	8.7	31.98	25.35	7.7	−12.1
	TAZ	2.50	2.55	2.7	8.9	15.00	17.03	8.1	5.5	35.00	31.59	6.0	−1.2

RE (%) calculated as relative error in comparison to fresh sample.

## Data Availability

The data presented in this study are available on request from the corresponding author.

## Supplementary Materials

The following supporting information can be downloaded at: https://www.mdpi.com/article/10.3390/ph17040526/s1, Figure S1: Optimization of the MS detection conditions; Figure S2: Optimization of the plasma sample pretreatment.; Figure S3: Selectivity investigation of the proposed CZE-MS/MS method; Figure S4: Illustrative extracted ion electropherograms obtained from the analysis of plasma QC samples at low concentration levels and the corresponding IS; Figure S5: Evaluation of the carry-over effect; Table S1: Recovery of the CZE-MS/MS method for β-lactam ATBs and inhibitors of β-lactamase in plasma QC samples.

## Abbreviations

ACN, acetonitrile; AGREE, Analytical GREEnness; AMP, ampicillin; AMX, amoxicillin; ATB, antibiotic; BAGI, Blue Applicability Grade Index; BGE, background electrolyte; CAZ, ceftazidime; CE, capillary electrophoresis; CTX, cefotaxime; CZE, capillary zone electrophoresis; DAD, diode array detector; DMSO, dimethyl sulfoxide; ESI, electrospray ionization; FDA, Food and Drug Administration; FLX, flucloxacillin; GAPI, Green Analytical Procedure Index; ICU, intensive care unit; IP, isopropyl alcohol; IS, internal standard; LC, liquid chromatography; LLOQ, lower limit of quantitation; LOD, limit of detection; MEKC, micellar electrokinetic chromatography; MeOH, methanol; MER, meropenem; MIC, minimal inhibitory concentration; MRM, Multiple Reaction Monitoring; MS, mass spectrometry; MS/MS, tandem mass spectrometry; NaOH, sodium hydroxide; (NH_4_)_2_CO_3_, ammonium carbonate; NH_4_HCO_3_, ammonium hydrogen carbonate; OXA, oxacillin; PIP, piperacillin; PK/PD, pharmacokinetic/pharmacodynamic; QC, quality control; QqQ, triple quadrupole; RE, relative error; RSD, relative standard deviation; S/N, signal-to-noise ratio; SIM, Selected Ion Monitoring; SL, sheath liquid; SUL, sulbactam; TAZ, tazobactam; TDM, therapeutic drug monitoring; TIC, total ion current; UV, ultraviolet.
