# Supplementary material for: Development and Validation of a Capillary Zone Electrophoresis–Tandem Mass Spectrometry Method for Simultaneous Quantification of Eight β-Lactam Antibiotics and Two β-Lactamase Inhibitors in Plasma Samples
Source: Pharmaceuticals (Basel). 2024 Apr 19;17(4):526. doi: 10.3390/ph17040526 (PMC11054939; doi:10.3390/ph17040526)
Supplement: Supplementary file 1 [file pharmaceuticals-17-00526-s001.zip › pharmaceuticals-2952184-supplementary.pdf]

pharmaceuticals

MDPI

Article

Development and Validation of a Capillary Zone Electrophoresis–Tandem Mass Spectrometry Method for Simultaneous Quantification of Eight β-Lactam Antibiotics and Two β-Lactamase Inhibitors in Plasma Samples

Ivana Cizmarova [1,2], Peter Mikus [1,2], Martin Svidrnoch [3] and Juraj Piestansky [2,4,*]

1 Department of Pharmaceutical Analysis and Nuclear Pharmacy, Faculty of Pharmacy, Comenius University in Bratislava, Odbojarov 10, SK-832 32 Bratislava, Slovakia; ivana.cizmarova@fpharm.uniba.sk (I.C.); mikus@fpharm.uniba.sk (P.M.)
2 Toxicological and Antidoping Center, Faculty of Pharmacy, Comenius University in Bratislava, Odbojarov 10, SK-832 32 Bratislava, Slovakia
3 AGEL Lab, Revolucni 2214/35, CZ-741 01 Novy Jicin, Czech Republic; martin.svidrnoch@lab.agel.cz
4 Department of Galenic Pharmacy, Faculty of Pharmacy, Comenius University in Bratislava, Odbojarov 10, SK-832 32 Bratislava, Slovakia
* Correspondence: piestansky@fpharm.uniba.sk

(Supplementary Material)

Table of content:

Figure S1: Optimization of the MS detection conditions.

Figure S2: Optimization of the plasma sample pretreatment.

Figure S3: Selectivity investigation of the proposed CZE-MS/MS method.

Figure S4: Illustrative extracted ion electropherograms obtained from the analysis of plasma QC samples at low concentration level and corresponding IS.

Figure S5. Evaluation of the carryover effect.

Table S1. Recovery of the CZE-MS/MS method for β-lactam ATBs, and inhibitors of β-lactamase in plasma QC samples.

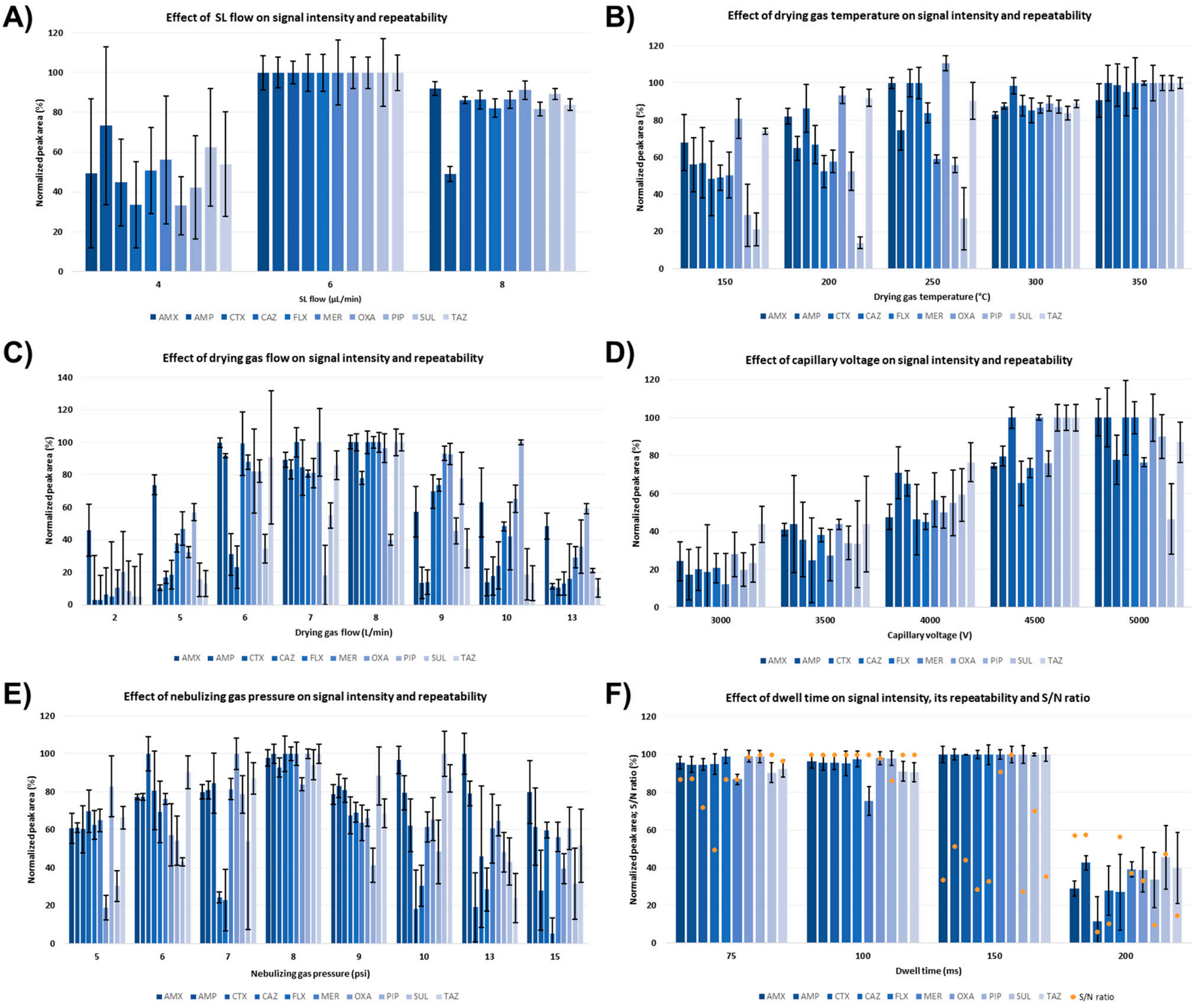

Figure S1. Optimization of the MS detection conditions. a) Effect of SL flow rate on the signal intensity and repeatability. b) Effect of drying gas temperature on the signal intensity and repeatability. c) Effect of drying gas flow rate on the signal intensity and repeatability. d) Effect of capillary voltage on the signal intensity and repeatability. e) Effect of nebulizing gas pressure on the signal intensity and repeatability. f) Effect of dwell time on the signal intensity and repeatability and the S/N ratio. The optimization procedure was performed with the use of ATBs standard solutions at the 10 µg. mL^{-1} concentration level.

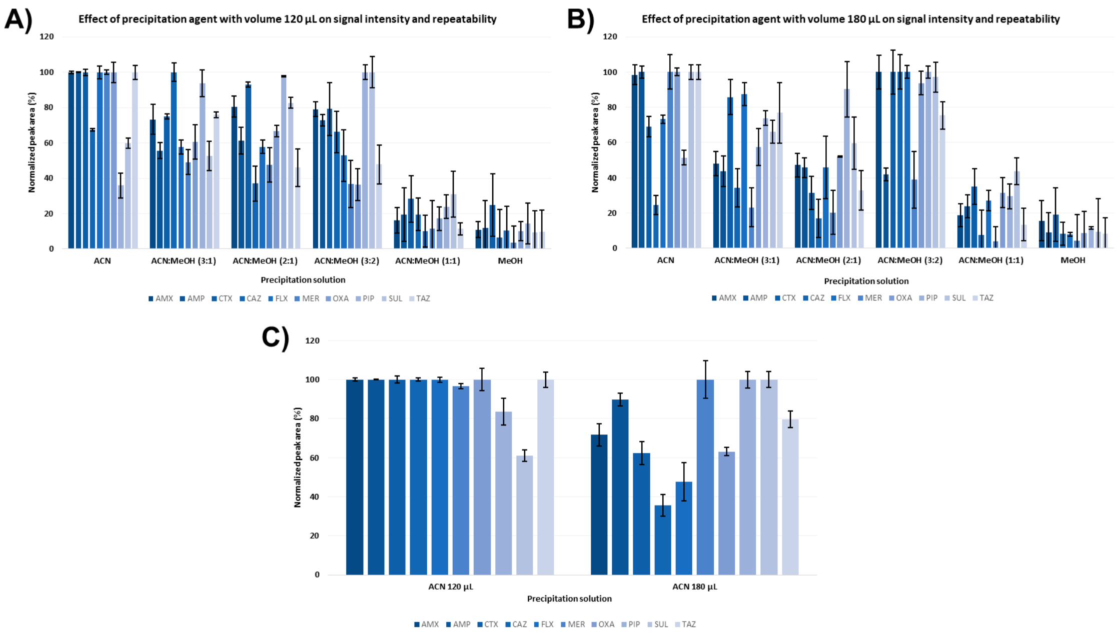

Figure S2. Optimization of the plasma sample pretreatment. a) Effect of precipitation agent composition on the signal intensity and repeatability. Tested volume 120 µL. b) Effect of precipitation agent composition on the signal intensity and repeatability. Tested volume 180 µL. c) Comparison of various volumes of ACN used as precipitation agent.

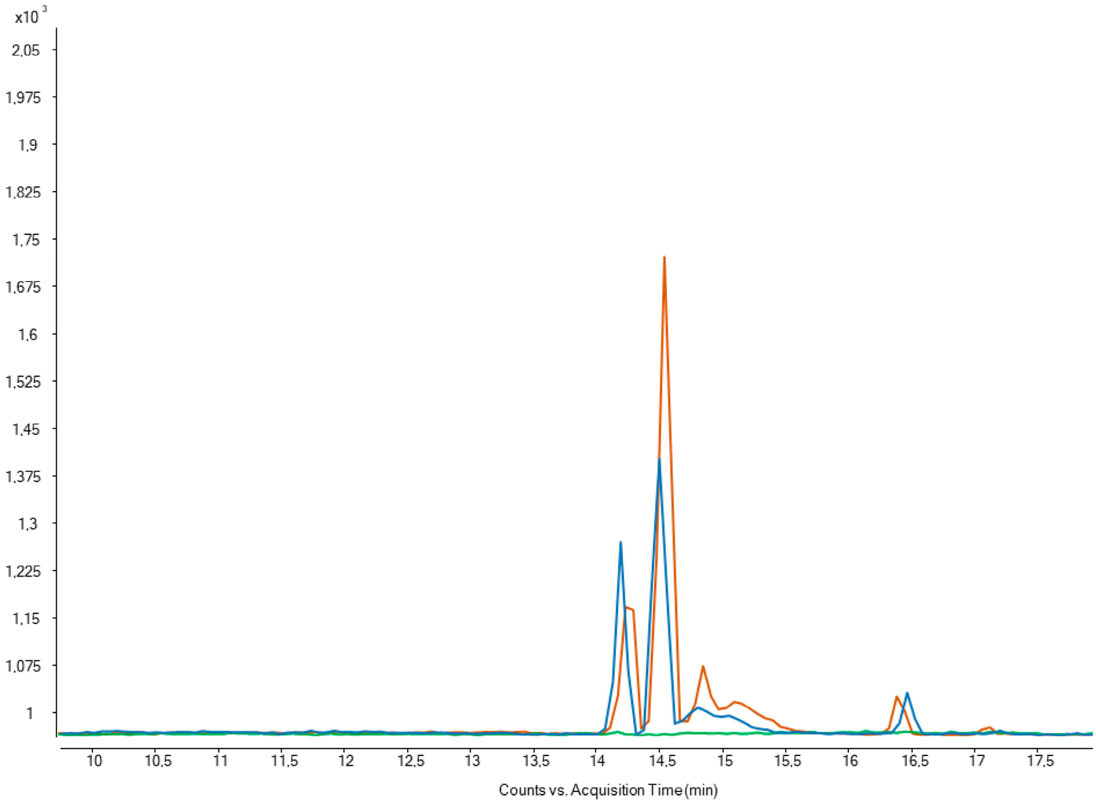

Figure S3. Selectivity investigation of the proposed CZE-MS/MS method. Illustrative total ion current (TIC) electropherograms obtained from the analysis of blank plasma sample (green), zero calibrator, i.e., blank plasma sample with IS (blue), and first calibrator, i.e., plasma samples spiked with the standards of the investigated substances at LLOQ level and IS (brown).

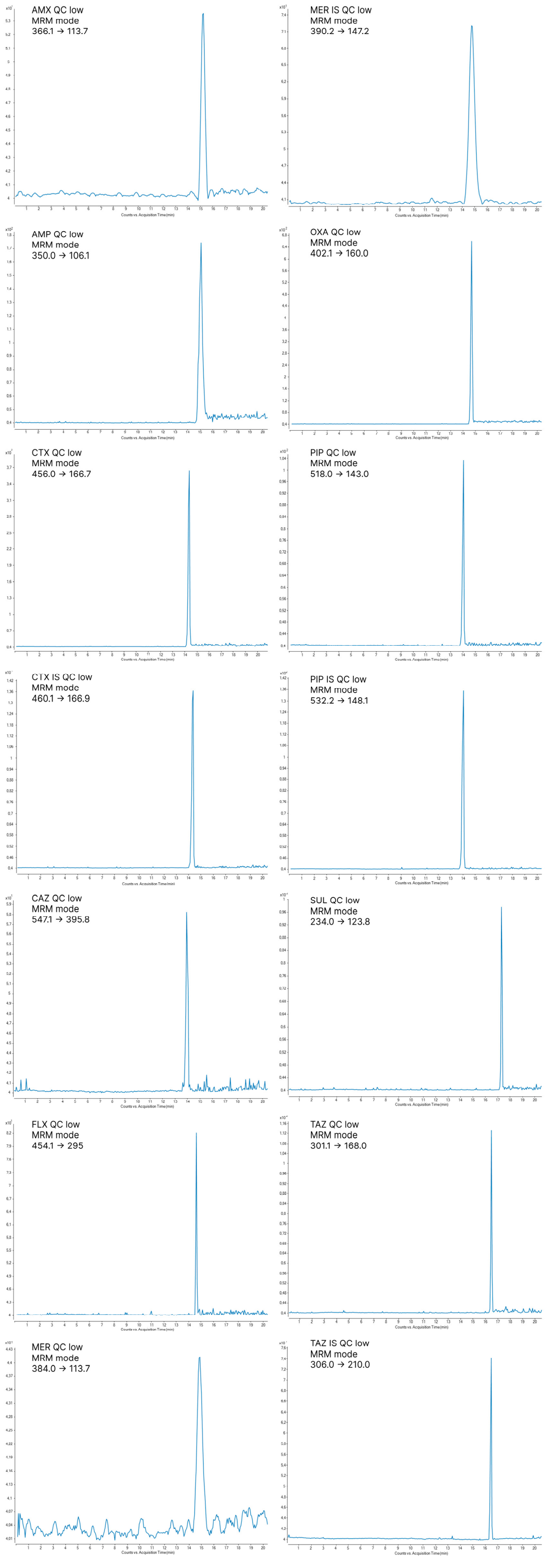

Figure S4. Illustrative extracted ion electropherograms obtained from the analysis of plasma QC samples at low concentration level and corresponding IS.

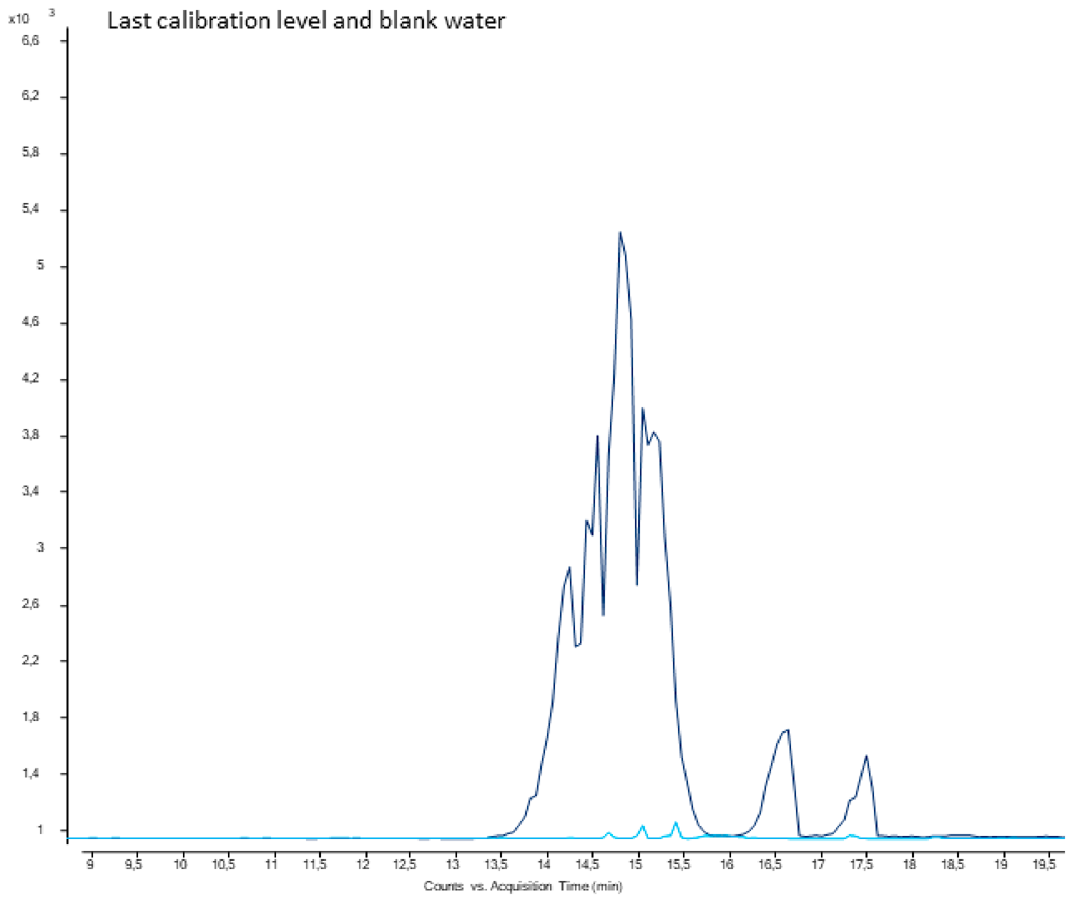

Figure S5. Evaluation of the carryover effect. Overlapped Total Ion Chromatogram (TIC) records from the analysis of the highest calibrator (dark blue) and water blank sample (sky-blue). The sample was injected hydrodynamically at a pressure of 50 mbar for 10 s, with an applied voltage of 20 kV.

Table S1. Recovery of the CZE-MS/MS method for β-lactam ATBs, and inhibitors of β-lactamase in plasma QC samples.

	Recovery (%)		
	**QC low**	**QC medium**	**QC high**
AMX	20	23	24
AMP	25	24	27
CTX	24	22	26
CAZ	29	20	20
FLX	20	22	22
MER	20	21	21
OXA	20	22	23
PIP	27	20	23
SUL	38	36	35
TAZ	37	40	36
